# Contraception determinants in youths of Sierra Leone are largely behavioral

**DOI:** 10.1186/s12978-018-0504-9

**Published:** 2018-04-19

**Authors:** Aline Labat, Marta Medina, Mohammed Elhassein, Afrina Karim, Mohammad B. Jalloh, Michèle Dramaix, Wei-Hong Zhang, Sophie Alexander, Kim E. Dickson

**Affiliations:** 1 Université Libre de Bruxelles, Ecole de Santé Publique, Research Center: Policies and Health Systems - International Health, 808, Route de Lennik, 1070 Brussels, Belgium; 2 hera, Right to Health and Development, Reet, Belgium; 3 FOCUS 1000, NGO, Freetown, Sierra Leone; 4 UNFPA, Freetown, Sierra Leone

**Keywords:** Adolescent, Youth, Behavior, Condoms, Contraception, Determinants, Gender, Health promotion, Sierra Leone

## Abstract

**Background:**

Sexual initiation occurs early in Sierra Leone. This study aims to analyze the determinants of condom and/or contraceptive use among a representative sample of young persons (10 to 24 years) in Sierra Leone.

**Methods:**

This is a secondary analysis of data from a study conducted to monitor the implementation of a UNFPA package of interventions directed to improve SRH in young people of Sierra Leone. This assessment was conducted in 2016 at the end of the Ebola outbreak. In consequence, determinants linked to healthy lifestyle behaviors and UNFPA interventions were explored in addition to the usual determinants: socio demographic and sexual lifestyle. This study is a household quantitative survey with open ended questions used to illustrate and complete the analysis.

**Results:**

A total of 1409 young people were interviewed: of these, 216 boys and 381 girls were sexually active. Those who were pregnant or wished for pregnancy were excluded, leaving 194 boys and 268 girls for the analysis of determinants. The proportion of young people using neither condom nor other contraception at their last sexual intercourse in the whole sample was 40.5% and there was no statistically significant difference between boys and girls (42.3 vs 39.2; *P* = 0.504). Determinants were assessed and, after multivariable analysis, results differed between boys and girls and showed the importance of behavioral aspects. Four determinants were common to boys and girls: literacy, distance, negotiation capacity and hand washing. However, the distance factor for girls was to the health facility and for boys it was to school. Three more determinants remained in the boy’s model: sleeping under a bednet, number of sexual partners and knowledge of contraceptive methods. Opinions about condoms and contraception revealed important barriers; opposition to contraceptive use was the main reason for non-use for both boys and girls, while lack of access was an important reason for boys.

**Conclusion:**

There is a need to reach out to the 40% of young people who are sexually active and neither pregnant nor with pregnancy desire, and are not using condom or contraception.

## Plain English summary

One of the most effective interventions to decrease maternal and child deaths is to ensure adequate access to family planning; and one of the most effective ways of avoiding the transmission of sexually transmitted diseases including HIV-AIDS is appropriate provision and use of condoms. However, access and use of both condoms and contraception is low in young people, and specifically in sub-Saharan Africa. To address the issue, this study explored what determined the use of contraception and condoms in a sample of young people between 10 and 24 years in Sierra Leone, through a population based survey. Of the 1409 respondents, 462 were sexually active, and neither pregnant nor wishing to be pregnant. More than 40% used neither condom nor contraception during their last sexual encounter. After multivariable analysis, four determinants were common to boys and girls: distance to school or the health facility, literacy, capacity to “negotiate” (asking not to have sex and asking for condom use), and hand washing. For boys only, three additional determinants remained: knowledge of contraceptive methods, number of lifetime sexual partners and sleeping under a bednet. Many non-users were opposed to condoms and contraception for subjective motives. In conclusion, specific actions are needed to reach out to this underserved population.

## Background

In sub-Saharan West Africa, maternal, neonatal and under-five-mortality remain high; key strategies to address this situation include increased family planning utilization and delayed first pregnancy [1]. Adolescent pregnancy in Sierra Leone is particularly frequent at an estimated 28% of all pregnancies [2], with 40% of maternal deaths occurring among adolescents [3, 4].

Two important issues of adolescent reproductive and sexual health are teenage pregnancy and risk of STIs HIV [5]. To avoid both, the safest option for sexually active unmarried youths is dual protection, and the least safe is neither. For women aged 15 to 24, the 2013 Sierra Leone demographic and health survey (SLDHS) showed condom use to be 1.0%, and any modern contraception to be 23% [2]. There is only limited information about the sexual and reproductive health (SRH) behaviors of younger adolescents (10–14 years), because in the DHSs, only adolescents above 15 years are interviewed.

Factors influencing youths’ utilization of condoms and contraception include inadequate sexual knowledge and risk perceptions, lack of skills and power to negotiate safer sex options [6].

The current study came about following the second of three planned population based surveys of young people. These were commissioned by UNFPA Sierra Leone, and performed by the consortium hera/Université Libre de Bruxelles/FOCUS 1000. The aim of the surveys was to monitor the implementation of the UNFPA component of a UK Department for International Development (DFID) funded project to “improve awareness of, access to, and uptake of, family planning, reproductive and maternal health services across Sierra Leone with a focus on young people”. This included improved access to family planning commodities, health provider training and community based interventions. Three community interventions were implemented, aimed at behavior change: (i) a national serial radio drama ‘Saliwansai’, (ii) the placement of Volunteer Peer Educators (VPEs) in communities, and, (iii) the enhancement of existing Community Wellness Advocacy Groups (CAGs). These interventions focused on topics such as delaying marriage, staying on at school, safe sex, or giving birth at a health facility. Because of the Ebola outbreack the third survey was not performed.

Beyond the monitoring aspects of the survey, available in the report [7], the aim of the present study was to better characterize determinants of utilization, in particular, behavioral aspects, as well as possible effect of gender or age. This assessment was conducted in 2016 at the end of the Ebola outbreak. In consequence, determinants linked to healthy lifestyle behaviors were also explored. The study was restricted to those interviewed youths who declared they were sexually active and neither they nor their partner was pregnant or wished to get pregnant. These were then classified into four mutually exclusive groups: dual protection, condom only, modern contraception other than condom, and non-users. The underlying assumption is that better characterization of “non-users” will contribute to better understanding and the development of targeted recommendations for further intervention.

## Methods

### Households survey

The monitoring survey was conducted in three provinces of Sierra Leone, deliberately excluding the Western Area with the capital Freetown, because it was known from the SLDHS that contraception and condom utilization was highest in this region.

Households were selected using a stratified three stage cluster sampling. The 149 chiefdoms in the three provinces were allocated to four strata representing the UNFPA intervention areas. In each chiefdom (primary sampling unit), four clusters of 25 households each, were selected with “Probability Proportional to Size”, and using the enumeration areas from the 2004 Sierra Leone Population and Housing Census. In total, the sample included 48 clusters. The sample size was computed, based on the expected time trend, between the planned first and last survey, for three indicators: contraceptive prevalence, condom use, and unmet need for family planning (FP).

A household questionnaire was administered to the head of the household, followed by an individual questionnaire to the males and females aged 10 to 24 years. For this questionnaire, in each household, a maximum of two persons were interviewed (one male and one female). Those of male sex are referred to as “boys” and those of female sex as “girls”. The household questionnaire described the composition and assets. The individual questionnaire included 74 items concerning: (1) socio-demographic characteristics; (2) SRH knowledge, attitude, and practice: sexual activity, negotiation, condom use, contraception; (3) exposure to UNFPA supported SRH promoting interventions and; (4) health and use of health services. Most of the questions were similar to those used in the DHS [8] and in the global school-based student health survey (GSHS) [9] to allow valid comparisons.

### Ethical considerations

Ethical approval was obtained from Ethical Research Committee of Université Libre de Bruxelles and by the Sierra Leone Ethics and Scientific Review Committee. Each head of household and each individual young person were asked whether they wished to participate. For those under 15 years of age, parental consent was also obtained.

### Variables for the study

Having sex with neither condom nor contraception was defined as: the last time the responder had sex, neither he/she, nor his/her partner used a condom or any modern method of contraception, and neither was pregnant nor wished to get pregnant.

Negotiation capacity was defined by combining two questions, which were different for boys and girls. For the girl the two questions were: (i) “can you ask the boy not to have sex” (consent), and (ii) “can you ask your partner to use a condom”. For the boys, the two questions were: (i) “is it acceptable if the girl asks not to have sex”, and (ii) “is it acceptable if the girl asks you to use a condom”. The two variables were combined and produced the following scale: 2 = responded yes to both questions; 1 = responded yes to one question only; and; 0 = responded no to both questions.

Literacy was defined by combining two questions: (i) “what was the highest year of studies you attained?” and, (ii), if they had responded “primary instruction” or “less” they were given a card with a sentence and asked to read it. The two variables were combined and produced the following scale: 1 = reads the entire sentence and/or has secondary, or higher education and; 0 = unable to read, whether the boy/girl had formal education or not.

Sexually active was used as an equivalent to “ever had sex”.

Contraception is used for “utilization of modern contraceptive commodities”.

“Condom or contraception use” is used for individuals using any of the following: (i) dual protection; (ii) condom alone; (iii) contraception alone.

In total 18 (19) determinants were explored. Nine determinants were socio demographic: age, province, two for distance (distance to school and distance to health facility), two for literacy-education, and three for status of household. In addition, two determinants reflected UNFPA interventions: exposure to enhanced CAGs and to VPEs; five (six) determinants indicated sexual lifestyle: negotiation, age at 1st sex, number of lifetime partners, parity (girls only), in union, and number of FP methods heard about; finally two determinants were considered to be markers of “healthy life style behaviors”: sleeping under a bednet and hand washing.

### Analysis

The primary outcome was “having sex with neither condom nor contraception” as defined above. All variables were categorical and were summarized with numbers and proportions. Pearson’s Chi^2^ test or Chi^2^ for trend where appropriate, were applied to compare proportions. Odds Ratios (OR) and their 95% Confidence Interval (CI) were used to measure the strength of the associations. Logistic models were built using a stepwise forward selection based on Wald’s test; all the considered independent variables except province were proposed for selection. The Hosmer and Lemeshow’s test was applied to the final models to check goodness of fit. Adjusted OR’s, their 95% CI and *P*-value from Wald’s test were derived from the final models. Significance level was 0.05 for all analyses and these were performed with STATA® v14.2.

Additionally, the data in responses and comments to open ended questions in the household survey questionnaire were used for illustration of the quantitative findings.

The full methodology for the survey is described in the reports of surveys 2014 and 2016 [7].

## Results

### Survey data

A total of 1172 heads of household agreed to participate in the survey and 1409 young people were interviewed, of which 587 (41.6%) reported they had ever had a sexual encounter. These heads of households were mainly farmers, half (49.3%) did not have access to improved water, and over a third had no access to a radio (37.6%) or a mobile phone (36.2%). In the 15 to 19 age group, 70% of girls and 45% of boys declared they were sexually active; while in the 10 to 14 year age group, it was respectively 8 and 4.5%).

The analysis pertains solely to the respondents who reported they had previously had sexual activity. It was considered acceptable to equate “sexually active” with “ever had sex”, because among those who reported ever having sex, 88.2% of boys and 72.4% of girls reported a sexual encounter in the last 3 months.

Respondents were assigned into five mutually exclusive groups based on the use of condom or contraception at the last sexual encounter: (i) dual protection (*n* = 70), (ii) condom alone (*n* = 21), (iii) contraception alone (*n* = 184), (iv) pregnant, or whose partner was pregnant or who wished to become pregnant (*n* = 125), and (v) neither contraception nor condom nor pregnancy desire (*n* = 187) (see Table 1).

**Table 1:** distribution of behaviors in relation to contraception in sexually active categorized into 5 mutually exclusive categories

Type of method used (“you or your partner”)	Sexually Active					
	Boys		Girls		Total	
	n	%	n	%	n	%
Dual	45	22%	25	7%	70	12%
Condom alone	12	6%	9	2%	21	4%
FP alone	55	27%	129	34%	184	31%
Pregnant or wishes to	12	6%	113	30%	125	21%
None of the above	82	40%	105	28%	187	32%
All	206	100%	381	100%	587	100%

This leaves for analysis of determinants, after exclusion of those pregnant or wishing to get pregnant, a sample of 462 young people: 187 who used neither condom nor modern contraception and 275 who used one or both.

Among the 125 pregnant or wishing to get pregnant, 52 were adolescents (10 to 19 years), 2 boys and 50 girls.

### Bivariate analysis of survey data

Our sample included 462 young people, 194 boys and 268 girls. The proportion of young people using neither condom nor contraception in the whole sample was 40.5%. There was no statistically significant difference between boys (42.3%) and girls (39.2%) (*P* = 0.504).

For boys (Table 2), the following characteristics were significantly associated with using a condom or contraception: province, distance to school, distance to health facility, education, literacy, meeting a VPE, capacity to negotiate, number of life time partners, number of FP methods heard about, sleeping under a bednet and hand washing.

**Table 2:** Determinants of having sex with neither condom nor contraception in boys: bivariate analysis

Variable (*n* = 194 unless otherwise specified)	Boys			
	n (%)	% neither condom nor contraception	Crude OR (95%CI)	P
Age (years)				0.519^a^
10–14	15 (7.7%)	46.7%	1.33 (0.44–4.00)	
15–19	96 (49.5%)	43.8%	1.18 (0.65–2.14)	
20–24	83 (42.8%)	39.8%	1	
Province				< 0.001
Eastern	61 (31.0%)	54.1%	6.33 (2.56–15.69)	
Northern	82 (42.3%)	50.0%	5.38 (2.25–12.83)	
Southern	51 (26.3%)	15.7%	1	
Distance to school (*n* = 191)				0.004
< 30 min	142 (74.3%)	35.9%	1	
≥ 30 min	49 (25.7%)	59.2%	2.59 (1.33–5.03)	
Distance to health facility (*n* = 191)				0.031
< 30 min	94 (49.2%)	34.0%	1	
≥ 30 min	97 (50.8%)	49.5%	1.90 (1.06–3.40)	
Education				< 0.001^a^
None	31 (16.0%)	71.0%	6.26 (2.54–15.44)	
Primary attended	74 (38.1%)	47.3%	2.30 (1.20–4.40)	
Primary completed or superior	89 (45.9%)	28.1%	1	
Literacy				< 0.001
Cannot read/only parts	47 (24.2%)	72.3%	5.39 (2.61–11.15)	
Able to read	147 (75.8%)	32.7%	1	
Source water* (*n* = 191)				0.058
Unimproved	92 (48.2%)	48.9%	1.75 (0.98–3.13)	
Improved	99 (51.8%)	35.4%	1	
Size of household (*n* = 193)				0.133
1–5	48 (24.8%)	45.8%	1.63 (0.79–3.37)	
6–8	85 (44.0%)	34.1%	1	
≥ 9	60 (31.0%)	50.0%	1.93 (0.98–3.80)	
Profession head (*n* = 191)				0.257
Farmer	120 (62.8%)	45.0%	1.42 (0.78–2.59)	
Other	71 (37.2%)	36.6%	1	
Met VPE				0.016
No	94 (48.5%)	51.1%	2.02 (1.14–3.61)	
Yes	100 (51.5%)	34.0%	1	
Met enhanced CAG				0.660
No	152 (78.4%)	41.5%	0.86 (0.43–1.70)	
Yes	42 (21.6%)	45.2%	1	
Negotiation (condom & refusal)				< 0.001^a^
Neither option	60 (30.9%)	60.0%	4.83 (2.31–10.12)	
One out of two options	58 (29.9%)	48.3%	3.01 (1.44–6.29)	
Both options	76 (39.2%)	23.7%	1	
Age at 1st sex (years)				0.340
< 15	69 (35.6%)	49.3%	1.55 (0.81–3.00)	
15	47 (24.2%)	38.3%	0.99 (0.47–2.09)	
16–24	78 (40.2%)	38.5%	1	
Lifetime partners (*n* = 192)				< 0.001
1	66 (34.4%)	53.0%	6.21 (2.54–15.20)	
2–4	74 (38.5%)	50.0%	5.50 (2.28–13.27)	
≥ 5	52 (27.1%)	15.4%	1	
In a union				0.666
Yes	49 (25.3%)	44.9%	1.15 (0.60–2.22)	
No	145 (74.7%)	41.4%	1	
Heard of FP methods				< 0.001
0–3	31 (15.9%)	80.7%	7.75 (3.00–20.00)	
4–8	163 84.0%)	35.0%	1	
Slept under bednet				< 0.001
Yes	123 (63.4%)	30.1%	1	
No	71 (36.6%)	63.4%	4.02 (2.17–7.46)	
Hand wash < 4 h				< 0.001
Yes	124 (63.9%)	26.6%	1	
No	70 (36.1%)	70.0%	6.43 (3.37–12.30)	

^a=^ chi^2^ for trend; VPE = Volunteer Peer Educator; CAG = Community wellness Advocacy Group; FP = Family Planning

*Improved water according to DHS definition

For girls, the following characteristics were significantly associated with using a condom or contraception: age, distance to health facility, education, literacy, capacity to negotiate (Table 3).

**Table 3:** Determinants of having sex with neither condom nor contraception in girls: bivariate analysis

Variable (*n* = 268 unless otherwise specified)			Girls	
	N (%)	% neither condom nor contraception	OR (95%CI)	P
Age (years)				0.022^a^
10–14	24 (9.0%)	62.5%	3.25 (1.30–8.09)	
15–19	129 (48.1%)	39.5%	1.27 (0.76–2.15)	
20–24	115 (42.9%)	33.9%	1	
Province				0.953
Eastern	72 (26.9%)	40.3%	1.10 (0.58–2.09)	
Northern	109 (40.7%)	39.5%	1.07 (0.60–1.90)	
Southern	87 (32.5%)	37.9%	1	
Distance to school				0.592
< 30 min	189 (70.5%)	40.2%	1	
≥ 30 min	79 (29.5%)	36.7%	0.86 (0.50–1.48)	
Distance to health facility				0.033
< 30 min	134 (50.0%)	32.8%	1	
≥ 30 min	134 (50.0%)	45.5%	1.71 (1.04–2.81)	
Education				0.023^a^
None	57 (21.3%)	54.4%	2.28 (1.18–4.43)	
Primary attended	109 (40.7%)	35.8%	1.07 (0.61–1.88)	
Primary or superior	102 (38.1%)	34.3%	1	
Literacy				< 0.001
Cannot read/ only parts	110 (41.0%)	54.6%	3.01 (1.81–5.02)	
Able to read	158 (59.0%)	28.5%	1	
Source water*				0.532
Unimproved	134 (50.0%)	41.0%	1.17 (0.72–1.91)	
Improved	134 (50.0%)	37.3%	1	
Size of household				0.613
1–5	95 (35.4%)	43.2%	1.30 (0.73–2.30)	
6–8	103 (38.4%)	36.9%	1	
≥ 9	70 (26.1%)	37.1%	1.01 (0.54–1.90)	
Profession head				0.333
Farmer	151 (56.3%)	41.7%	1.28 (0.78–2.10)	
Other	117 (43.7%)	35.9%	1	
Met VPE				0.059
No	139 (51.9%)	44.6%	1.61 (0.98–2.64)	
Yes	129 (48.1%)	33.3%	1	
Met enhanced CAG				0.623
No	153 (57.1%)	37.9%	0.88 (0.54–1.45)	
Yes	115 (42.9%)	40.9%	1	
Negotiation (condom & refusal)				< 0.001^a^
Neither option	86 (32.1%)	61.6%	6.78 (3.46–13.29)	
One out of two options	88 (32.8%)	38.6%	2.66 (1.36–5.19)	
Both options	94 (35.1%)	19.2%	1	
Age at 1st sex (years) (*n* = 266)				0.116
< 15	131 (49.2%)	43.5%	1.84 (1.01–3.35)	
15	57 (21.4%)	42.1%	1.74 (0.85–3.56)	
16–24	78 (29.3%)	29.5%	1	
Lifetime partners (*n* = 266)				0.353
1	140 (52.6%)	43.6%	1.39 (0.44–4.36)	
2–4	112 (42.1%)	34.8%	0.96 (0.30–3.07)	
≥ 5	14 (5.3%)	35.7%	1	
Parity				0.573
0	139 (51.9%)	38.1%	1	
1	81 (30.2%)	37.0%	0.95 (0.54–1.68)	
≥ 2	48 (17.9%)	45.8%	1.37 (0.71–2.66)	
In a union				0.335
Yes	128 (47.8%)	42.2%	1.27 (0.78–2.08)	
No	140 (52.2%)	36.4%	1	
Heard of FP methods				0.155
0–3	27 (10.1%)	51.9%	1.78 (0.80–3.95)	
4–8	241 (89.9%)	37.8%	1	
Slept under bednet				0.654
Yes	203 (75.7%)	38.4%	1	
No	65 (24.3%)	41.5%	1.14 (0.64–2.01)	
Hand wash < 4 h				0.033
Yes	214 (79.9%)	36.0%	1	
No	54 (20.1%)	51.9%	1.91 (1.05–3.50)	

^a=^ chi^2^ for trend; HF = health facility, VPE = Volunteer Peer Educator; CAG = Community Wellness Advocacy Group

*Improved water according to DHS definition

Statistically significant interactions were observed between sex of the respondent (boy or girl) and five of the determinants: province, distance from school, number of FP methods heard about, having slept under a bednet and having washed hands in the previous 4 h. For all these variables, the association with the outcome was stronger in boys than in girls.

### Multivariable analysis of survey data

A multivariable model has been developed for boys and girls; results are presented in Tables 4 and 5.

**Table 4:** Multivariable analysis of utilization of condom/contraception at last intercourse: 189 sexually active boys

Variable (in order of inclusion)	Adjusted OR (95%CI)	P
Hand wash < 4 h		< 0.001
Yes	1	
No	6.57 (2.63–16.45)	
Literacy		< 0.001
Cannot read/only parts	8.50 (2.90–24.94)	
Able to read	1	
Heard of FP methods		0.018
0–3	4.37 (1.28–14.88)	
4–8	1	
Lifetime partners		0.004
1	5.86 (1.59–21.70)	
2–4	9.55 (2.51–36.36)	
≥ 5	1	
Distance to school		0.004
< 30 min	1	
≥ 30 min	5.16 (1.71–15.55)	
Slept under bednet		0.009
Yes	1	
No	3.27 (1.35–7.91)	
Negotiation (condom & refusal)		0.030
Neither option	3.81 (1.37–10.62)	
One out of two options	2.50 (0.90–6.91)	
Both options	1	

H-L test: *P* = 0.684 – Pseudo R^2^ = 0.435; FP = Family Planning

Not included (NS): age, age 1st sex, distance to HF, education, union, met VPE, met CAG, type water, size household, occupation head household

**Table 5:** Multivariable analysis of utilization of condom/contraception at last intercourse: 268 sexually active girls

Variable (in order of inclusion)	Adjusted OR (IC95%)	P
Negotiation (condom & refusal)		< 0.001
Neither option	6.88 (3.34–14.20)	
One out of two options	2.73 (1.35–5.50)	
Both options	1	
Literacy		0.004
Cannot read / only parts	2.28 (1.30–3.99)	
Able to read	1	
Distance to health facility)		0.008
< 30 min	1	
≥ 30 min	2.16 (1.23–3.79)	
Hand wash < 4 h		0.047
Yes	1	
No	2.00 (1.01–3.98)	

H-L test: *P* = 0.846 – Pseudo R^2^ = 0.167; FP = Family Planning

Not included (NS): age, age 1st sex, distance to school, education, number of partners, union, heard PF methods, met VPE, met CAG, parity, type water, size household, occupation head household, slept under bednet

Only four variables remained in the girls’ model, whereas seven remained in the boys’. Three variables were common to the girls’ and boys’ models: hand washing in the previous 4 h, literacy and capacity to negotiate. However, in boys, hand washing in previous 4 h was the first variable to enter the model, while it was negotiation for the girls’ model. Literacy was the second variable to enter in both models; however the corresponding adjusted OR was higher in the boys’ model. Distance appears in both models, though it is to the health facility for girls and to school for boys. In addition to these four variables, in the boys’ model, number of partners, number of FP methods heard about, and sleeping under a bednet remained significant after adjusting for all other included in the model.

Except for literacy and distance, all other determinants are behavioral, whereas the more classic determinants like economic assets or occupation do not remain in the multivariable model.

Additionally, we explored reasons given for not using a condom or contraception (Table 6). All reasons which were given by more than 5% of respondents for condoms, and by more than 15% for contraception are presented.

**Table 6:** Reasons given for not using condom or contraception

Most frequent reasons in boys (*n* = 79)		Most frequent reasons in girls (*n* = 104)	
	n (%)		n (%)
For not using condom			
1.Partner doesn’t want	37 (46.8%)	1.Partner doesn’t want	73 (70.2%)
2.Condoms not available	31 (39.2%)	2.Faithful	39 (37.5%)
3.Don’t know where to get	25 (31.7%)	3.Condoms not available	25 (24.0%)
4.Faithful	22 (27.9%)	4.Don’t know where to get	16 (15.4%)
5.Religious	21 (26.6%)	5.Religious	13 (12.5%)
For not using contraception			
1.Partner opposed	33 (41.8%)	1.Partner opposed	40 (38.5%)
2.Not married	18 (22.8%)	2.Others opposed	25 (24.0%)
3.Lack of access	17 (21.5%)	3.Knows no method	18 (17.3%)
4.Respondent opposed	15 (19.0%)	4.Knows no source	18 (17.3%)
5.Knows no method	14 (17.7%)	5.Respondent opposed	16 (15.4%)
6.Knows no source	14 (17.7%)	6.Religious	14 (13.5%)

Three boys and one girl did not answer

Opposition of the partner was the most frequent reason for not using condom or contraception for both boys (46.8%) and girls (70.2%). Interestingly, both boys and girls put the responsibility of the opposition on their partner. Non availability of condoms was also among the top reasons mentioned. Lack of access to contraception was one of the top three mentioned by boys but not by girls.

### Comments made by youths in the individual survey

Some of the comments about not using condoms or modern contraception, in open ended questions in the household survey, warrant further thought from planners and educators.

Two girls explained that they had used neither condom nor family planning because their previous intercourse had been a rape (2 out of 105 non-users). Eight boys and girls believed the condom constituted a health risk: one believed it could kill, one other that it brings on “death and illness”, one mentioned it brought on infection, three mentioned it was harmful for health, one girl mentioned that it would corrupt her milk, and two more were worried that it would stick in the vagina. Further comments included: “shameful”, “forbidden”, “inconvenient”, “lack of knowledge about utilization”, and “waste of time”.

## Discussion

The overall contraceptive prevalence rate (condom alone, or contraception alone or both) was 55% for boys and 43% for girls. Dual protection was observed in a higher number of boys (22%) than girls (7%). In addition, globally 6% of boys and 30% of girls declared that their partners or themselves were pregnant or wished to be. Among these, 42% (17% of the boys and 44% of the girls) were in the 10–19 age group, which is in opposition with the National Strategy for the reduction of teen-age pregnancy [10].

In the multivariable model, four determinants of condom or contraception use were common to boys and girls: literacy, distance, negotiation and hand washing. The distance which remained in the model for girls was to the health facility, while for boys it was to school. This could be due to the fact that girls most commonly obtain contraception from health providers at facilities while with boys this is not critical to access condoms.

In the bivariate model there was significant gender interaction for five determinants of condom or contraception use. One of the two UNFPA interventions, the VPE placements was a determinant for boys and there was a strong trend for girls. The enhanced CAGs on the other hand showed no effect. However, there are various possible explanations: (i) the likelihood of meeting a VPE is higher than for a CAG; (ii) in general, the core content of VPE messages is more related to sexual health; and (iii) CAG messages may have been more modulated in relation to the EVD outbreak, with a greater focus on prevention of infection dissemination and hand washing in particular. This hypothesized change may be reflected in the strong association with hand washing found for both boys and girls.

The open questions show serious misgivings about condoms and contraception. Two girls explained the motive for non use as being raped; though this is not numerically important this needs to be kept in mind.

The study has many strengths. Data were collected with electronic tablets which enhanced the quality and the completeness of data. It has been suggested that electronic tablets might be one of the greatest advances of the 21st century, because they contribute to good monitoring in Low and Middle Income Countries (LMICs) [11]. Boys and girls answered the same questions, including questions about barriers or negotiation, this has allowed to show strong gender differences. The behavioral determinants showed significant gender interaction, with effects systematically stronger in boys. In a recent UK study, gender and age were determinants of contraception intention, supporting the need for tailored sex education [12].

Using, to a large extent, the same questions as in the DHS and the GSHS allows for comparisons with these data sets. Regarding descriptive data such as economic assets, distance or education the fact that the results of this survey are essentially in the same direction and of the same order of magnitude as the results from the 2013 SLDHS provides a certain degree of data validation.

The study also has some weaknesses: the sample size is small, especially for sexually active youngsters of less than 15, decreasing power, and the data pertain to the post EVD outbreak in Sierra Leone, decreasing external validity.

A number of the study results are similar to published results from other countries; however this study provides unique information for the Sierra Leone context. Determinants which remain in the final models both for boys and girls: literacy and distance to school and health facilities are well described in four recent systematic reviews of contraception use determinants in LMICs in general [13] or specifically in sub-Saharan Africa [14–16]. Distance presumably functions in two manners: directly as the time it takes in terms of reduced access, and probably also as an indicator of populations difficult to reach out to, with less opening to the modern world. Literacy remained in the present model; whereas age and education, which are ubiquitous in studies of determinants of contraception use, and which were present in the bivariate analysis, did not remain in the multivariate. A possible explanation is that literacy is a direct resultant of both age and exposure to education and might be a better reflection of the causal pathway of non-utilization of contraception. An argument in this direction can be found in a quasi-experimental study; Leon et al. implemented, in India, a three-year community intervention to globally promote woman’s empowerment. They observed, in the intervention area, a significant change on beliefs about the decision to have children, but only in the illiterate women [17]. The results of Leon generate the hypothesis that literacy acts not only as a determinant of utilization, but possibly needs to be taken into account for choice of interventions.

Two other determinants were common to boys and girls and are essentially behavioral: the first is related to sexual behavior and includes a composite of “sexual consent” and “condom negotiation”. This determinant is not found in many studies. However, in a qualitative study in young high school girls in Ghana, all who were using condoms considered they were able to require their partner to use it [18].

Two more behavioral determinants warrant some discussion, hand washing, which remained in both the girls’ and boys’ model, and bednet utilization which remained for boys only. Unsurprisingly, no data on contraception and either hand washing, or bednet utilization, could be found in the literature. However it can be hypothesized that determinants could be similar, as all of these are desirable behaviors. Two such examples follow. A study in Nigeria on school children assessed which canals were most effective in increasing hand washing during the EVD outbreak; the two most effective were the church and television, the second being once more, a marker of more affluent households [19]. For bednet utilization, in a recent survey of determinants in Cameroon, knowledge of utility and educational level remain in the final model suggesting again, that these are more global determinants of healthy behavior [20]. Two more determinants were specific to boys only: theoretical knowledge of contraception, and number of partners, which appear in most systematic reviews such as those mentioned above.

The open questions revealed, among others, two issues: coerced sex in teen-agers and severe misconceptions about condoms and contraception. Both these are documented in previously published studies, as in deprived populations of Kampala for coercion where 25% of the interviewed had been raped [21] or urban Cameroon for misconceptions about contraception [22].

This study has direct implications. The first two pertain to the content of SRH messages for adolescents. Should there be a shift towards a clear recommendation of “dual protection” or “safe sex”? A stronger emphasis on promotion of dual protection for young, unmarried people would be beneficial, even in a country with a relatively low HIV prevalence like Sierra Leone (1.5% of 15–49 years), to avoid other forms of STIs (13.4% for males and 10.5% for females), self reported, in the same age group [2].

The other major implication is about reaching out to underserved populations. In a study in Senegal, on “harder to reach” populations, these are defined as “less exposed to external influence and at risk of being underserved despite their high level of need”, and considered to belong to the three categories: adolescents, unmarried and rural poor [23]. Changing attitudes in communities which are far from everything is a true challenge [24].

## Conclusions

This study shows that sexually active young people who use condoms or contraception are generally better educated, easier to reach and have safer health behaviors.

Our study reinforces the notion that interventions need to be targeted specifically, taking into account characteristics such as gender, age, marital status, literacy and outreach issues.

This study suggests that a truly comprehensive vision of sexual health is warranted, including topics apparently not systematically addressed at present, such as coercion, intra partner violence, negotiation, consent, and avoiding teen-age pregnancy. This can theoretically be best addressed through school programs such as the PREPARE in South Africa [25].

## Abbreviations

CAG: Community Wellness Advocacy Groups
CI: Confidence interval
DFID: Department for International Development (UK AID)
DHS: Demographic and Health Survey
EVD: Ebola virus disease
FP: Family planning
GSHS: Global School-based student Health Survey
HIV-AIDS: Human Immunodeficiency Virus - Acquired Immune Deficiency Syndrome
IRMNH: Improving Reproductive Maternal and Newborn Health
IUD: Intrauterine device
LMIC: Low and middle income countries
OR: Odds ratios
SLDHS: Sierra Leone Demographic and Health Survey
SRH: sexual and reproductive health
STI: Sexually Transmitted Infection
UNFPA: United Nations Population Fund
UNICEF: United Nations International Children’s Emergency Fund
VPE: Volunteer Peer Educators
